# KIF9 Ameliorates Neuropathology and Cognitive Dysfunction by Promoting Macroautophagy in a Mouse Model of Alzheimer's Disease

**DOI:** 10.1111/acel.14490

**Published:** 2025-01-19

**Authors:** Maoju Wang, Song Guo, Lilin Yi, Zhaolun Li, Xiuyu Shi, YePeng Fan, Man Luo, Yan He, Weihong Song, Yehong Du, Zhifang Dong

**Affiliations:** ^1^ Growth, Development, and Mental Health of Children and Adolescence Center, Pediatric Research Institute, Ministry of Education Key Laboratory of Child Development and Disorders, National Clinical Research Center for Child Health and Disorders, Chongqing Key Laboratory of Child Neurodevelopment and Cognitive Disorders Children's Hospital of Chongqing Medical University Chongqing China; ^2^ Townsend Family Laboratories, Department of Psychiatry University of British Columbia Vancouver British Columbia Canada; ^3^ Oujiang Laboratory (Zhejiang Lab for Regenerative Medicine, Vision and Brain Health), Institute of Aging, Key Laboratory of Alzheimer's Disease of Zhejiang Province, Zhejiang Clinical Research Center for Mental Disorders, School of Mental Health and the Affiliated Kangning Hospital Wenzhou Medical University Wenzhou Zhejiang China

**Keywords:** Alzheimer's disease, KIF9, macroautophagy, memory

## Abstract

Alzheimer's disease (AD) is a prevalent neurodegenerative disorder affecting the elderly. The imbalance of protein production and degradation processes leads to the accumulation of misfolded and abnormally aggregated amyloid‐beta (Aβ) in the extracellular space and forms senile plaques, which constitute one of the most critical pathological hallmarks of AD. KIF9, a member of the kinesin protein superfamily, mediates the anterograde transport of intracellular cargo along microtubules. However, the exact role of KIF9 in AD pathogenesis remains largely elusive. In this study, we reported that the expression of kinesin family member 9 (KIF9) in the hippocampus of APP23/PS45 double‐transgenic AD model mice declined in an age‐dependent manner, concurrent with macroautophagy dysfunction. Furthermore, we found that KIF9 mediated the transport of lysosomes through kinesin light chain 1 (KLC1), thereby participating in the degradation of amyloidogenic pathway‐related proteins of Aβ precursor protein (APP) in AD model cells through promoting the macroautophagy pathway. Importantly, genetic upregulation of KIF9 via adeno‐associated virus (AAV) diminished Aβ deposition and alleviated cognitive impairments in AD model mice by enhancing macroautophagy function. Collectively, our findings underscore the ability of KIF9 to promote macroautophagy through KLC1‐mediated anterograde transport of lysosomes, effectively ameliorating cognitive dysfunction in AD model mice. These discoveries suggest that KIF9 may represent a novel therapeutic target for the treatment of AD.

## Graphical abstract

KIF9 is downregulated in AD, accompanied by dysfunction in macroautophagy. Genetic upregulation of KIF9 enhances macroautophagy by promoting KLC1‐mediated anterograde transport of lysosomes, which effectively reduces Aβ deposition and alleviates cognitive impairments in AD.

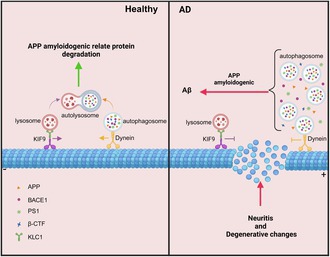

## 1 Introduction

Alzheimer's disease (AD) is a neurodegenerative disorder that can be either hereditary or sporadic (Knopman et al. 2021; Luo et al. 2024) and represents the most common form of dementia in the elderly (“2023 Alzheimer's disease facts and figures” 2023; Peric and Annaert 2015). The disease is characterized by the accumulation of misfolded and aggregated proteins, notably extracellular amyloid‐beta (Aβ) plaques and intracellular neurofibrillary tangles (NFTs) formed by hyperphosphorylated tau proteins (Liu et al. 2024). Due to these aberrant protein aggregations, AD is often referred to as a proteinopathy (Boland et al. 2018). Although the precise pathogenic mechanisms remain incompletely understood, increasing evidence suggests that dysfunction in the autophagy–lysosome pathway may precede the formation of these pathological features (Long et al. 2020, 2024; Zare‐Shahabadi et al. 2015), contributing to the aggregation and spread of neurotoxic protein oligomers, ultimately leading to neuronal loss and dementia (Harris and Rubinsztein 2011; Joe and Ringman 2019).

Macroautophagy, a lysosome‐mediated autophagy process, is a crucial mechanism for maintaining cellular homeostasis, especially within neurons, where it represents an important and conserved degradation pathway (Rubinsztein, Codogno, and Levine 2012). Autophagosomes are responsible for transporting abnormally aggregated proteins and damaged organelles to lysosomes for degradation (Cai et al. 2016). In healthy neural tissues, mature autophagosomes are rapidly transported to the cell body via the microtubule system through dynein ‐ mediated retrograde transport, where they fuse with lysosomes to form autolysosomes (Jahreiss, Menzies, and Rubinsztein 2008). Simultaneously, lysosomes, primarily located around the nucleus, also move along microtubules in both anterograde and retrograde directions between the cell center and periphery under the guidance of kinesin and dynein (Pu et al. 2016; Roney et al. 2021). Thus, the stability of intracellular microtubule function is essential for the normal transport and fusion of autophagosomes with lysosomes. However, under the pathological conditions of AD, neuritic changes and senile plaques lead to significant alterations in the neuronal cytoskeleton, often causing axonal damage before synaptic loss (Sanchez‐Varo et al. 2012). For instance, Suzuki and Terry initially observed numerous round or oval vesicles, approximately 0.5 μm in diameter, in dystrophic neurites of AD brains (Suzuki and Terry 1967). It was not until 2005 that Nixon and colleagues used electron microscopy to definitively identify these vesicles as autophagic vacuoles (Nixon et al. 2005). In AD brains, macroautophagy functionality is compromised (Banerjee, Beal, and Thomas 2010). Therefore, promoting the effective transport of autophagosomes and lysosomes could enhance fusion, reduce autophagosome accumulation, and inhibit the aggregation and spread of neurotoxic proteins, potentially offering a strategy for treating AD.

The kinesin superfamily consists of 45 known kinesins (Yildiz and Selvin 2005), classified into three types based on the location of their motor domains: NH2‐terminal motor domain type (N‐kinesins) (Hirokawa et al. 1989), middle motor domain type (M‐kinesins) (Noda et al. 1995), and COOH‐terminal motor domain type (C‐kinesins) (Yang, Laymon, and Goldstein 1989). Among these, 39 belong to N‐kinesins and move anterogradely along microtubules (Meluh and Rose 1990). Kinesins are essential molecular motor proteins required for microtubule‐dependent transport in neurons. During macroautophagy, they mediate the anterograde movement of lysosomes. For instance, the heavy chains of kinesin‐1 and kinesin‐3 interact with the small GTPase Arl8b and its effector SKIP through the kinesin light chain 1 (KLC1) to form complexes. These complexes then bind to BORC, which is anchored to lysosomes, to facilitate anterograde lysosomal transport (Farías et al. 2017). Kinesin‐1 dysfunction impairs the anterograde transport of lysosomes to axons, affecting fusion and maturation with autophagosomes and leading to diseases such as Niemann‐Pick disease (Roney et al. 2021). On the contrary, the overexpression of kinesin‐1 can enhance the anterograde transport of lysosomes and mitigate the axonal transport impairment of lysosomes induced by trimethyltin chloride, thereby promoting autophagy (Liu et al. 2021). In addition, a recent study has shown that KIF5B is involved in lysosome transport during classical swine fever virus infection by co‐localizing KIF5B with Rab7, Rab11, or Lamp1 (Lou et al. 2023). Thus, kinesins play a critical role in lysosomal transport along microtubules and are implicated in neurodegenerative diseases related to axonal transport (Hayashi and Sasaki 2023).

Kinesin family member 9 (KIF9), an N‐kinesin subtype within the kinesin superfamily, primarily performs anterograde movement on microtubules (Miki et al. 2001). KIF9 was initially studied for its interaction with the GTP‐binding protein GEM (Piddini et al. 2001) and has been reported to regulate the flagellar movement of mouse sperm via the central pair microtubule (Miyata et al. 2020), control ciliary beating and motile complex localization in multiciliated cells (Konjikusic et al. 2023), and interact with reggie/flotillin proteins to regulate matrix degradation in macrophage podosomes (Cornfine et al. 2011). Structurally, KIF9, like kinesin‐1, mediates anterograde cargo transport along microtubules. However, research on the role of KIF9 in lysosomal transport and the macroautophagy pathway is limited, and its involvement in neurodegenerative diseases, particularly AD, is even less explored. Therefore, investigating KIF9 and its potential interactions in AD could provide new insights into the disease's pathogenesis and reveal novel therapeutic targets.

In this study, we found that the expression of KIF9 significantly decreased in an age‐dependent manner in the hippocampus of AD model mice, accompanied by pronounced macroautophagy dysfunction. Next, we revealed that KIF9 mediated facilitated lysosomal transport through its light chain KLC1, thereby promoting the degradation of Aβ precursor protein (APP) amyloidogenic pathway‐related proteins via the macroautophagy pathway in AD model cells. Moreover, overexpression of KIF9 via AAV_KIF9_ markedly enhanced macroautophagy and improved cognitive dysfunction in AD model mice.

## 2 Materials and Methods

### 2.1 Animals

APP23/PS45 transgenic mice were housed in a specific pathogen‐free environment at the Animal Care Center of Children's Hospital of Chongqing Medical University. The mice were kept under a 12‐h light/dark cycle (7 am to 7 pm) with controlled temperature and humidity. They had ad libitum access to water and food. All animal procedures were conducted in accordance with the guidelines established by the Chongqing Science and Technology Commission and were approved by the Animal Ethics Committee of Children's Hospital of Chongqing Medical University.

### 2.2 Antibodies

The anti‐KIF9 antibody (1:200, AP5953b) was purchased from Abcepta (Suzhou, Jiangsu, China). The anti‐P62 antibody (1:1000, H00008878‐M01), anti‐ubiquitin antibody (1:1000, #10201‐2‐AP), and anti‐GAPDH antibody (1:5000, ARG10112) were obtained from Abnova (Taipei, Taiwan, China), Proteintech (Wuhan, Hubei, China), and Arigo Biolaboratories Corp (Taiwan, China), respectively. The anti‐LC3 antibody (1:1000, #12741) and anti‐Lamp1 antibody (1:1000, #9091) were purchased from Cell Signaling Technology (Danvers, MA, USA). The anti‐BACE1 antibody (1:1000, ab108391) and anti‐PS1 antibody (1:1000, ab76083) were obtained from Abcam (Cambridge, MA, USA). The C20 antibody (1:1000) for detecting APP and its CTFs was provided by Professor Weihong Song.

### 2.3 Cell Culture, Transfection, and Treatment

2EB2 cells are HEK293 cells stably transfected with the human Swedish mutants APP695 and BACE1, obtained from Professor Weihong Song's laboratory. HEK293 cells were cultured in a 37°C incubator with 5% CO_2_ in a medium consisting of 90% Dulbecco's modified Eagle's medium (DMEM) (Gibco, New York, USA) and 10% fetal bovine serum (FBS) (Gibco, New York, USA). For 2EB2 cells, the medium also contained 25 μg/mL G418 (Gibco, New York, USA) and 100 μg/mL Zeocin (Invitrogen, USA).

Cells were seeded in six‐well plates (Corning, USA), and once they reached 70%–80% confluence, transfections were carried out using PEI (24765‐100, Ploysciences, USA) with the KIF9 plasmid (EX‐Z6499‐M91, GeneCopoeia, USA) or its empty vector. Transfections were performed according to the protocol provided in the PEI manual and maintained for 24 h.

To inhibit autophagosome–lysosome fusion without affecting lysosomal acidity and/or degradation activity (Mauthe et al. 2018), cells were treated with 50 μM chloroquine (CQ) (Sigma, St. Louis, MO, USA) for 24 h. Additionally, for investigating the effect of KIF9 overexpression on protein degradation through lysosomes, cells were transfected with the KIF9 plasmid for 4 h, followed by treatment with 50 μM CQ for an additional 24 h.

### 2.4 Autophagy Flux Detection

HEK293 and 2EB2 cells were seeded into 24‐well plates and transfected with the mRFP‐GFP‐LC3 plasmid (a gift from Professor Weihong Song) for 24 h. For the 2EB2 cells, the KIF9 overexpression plasmid or its negative control plasmid was transfected for 4 h, followed by transfection with the mRFP‐GFP‐LC3 plasmid for an additional 24 h. The cells were then fixed with 4% paraformaldehyde (PFA) and mounted with DAPI solution (Vector Laboratories, Burlingame, USA). Images were captured using a confocal microscope (Nikon, Tokyo, Japan). Autophagic flux was assessed by evaluating the number of GFP and mRFP dots per cell. Co‐localization of GFP and mRFP, observed as yellow dots, indicates autophagosomes, while free red dots represent autolysosomes.

### 2.5 Quantitative Real‐Time PCR (Q‐PCR)

Total RNA was extracted from cells using TRIzol reagent (Takara, Otsu, Shiga, Japan). RNA concentration and purity were determined using a NanoDrop 2000 spectrophotometer (NanoDrop Technologies, Wilmington, DE, USA). First‐strand complementary DNA (cDNA) synthesis was performed using 1 μg of RNA with the PrimeScript RT kit (Takara, Otsu, Shiga, Japan). Q‐PCR was conducted to detect cDNA levels of APP, BACE1, and PS‐1 using the CFX Manager software detection system (Bio‐Rad). Primer sequences were as follows: APP (forward: 5′‐ATGCCGTTGACAAGTATCTCG, reverse: 5′‐TCTGCCTCTTCCCATTCTCTC); BACE1 (forward: 5′‐TACCAACCAGTCCTTCCGC, reverse: 5′‐CTCCCATAACAGTGCCCGT); PS1 (forward: 5′‐ATGCCGTTGACAAGTATCTCG, reverse: 5′‐TCTGCCTCTTCCCATTCTCTC) and GAPDH (forward: 5′‐AACTGCTTAGCACCCCTGGC, reverse: 5′‐ATGACCTTGCCCACAGCCTT).

### 2.6 Immunocytochemical Staining

After seeding 2EB2 cells into 12‐well plates and allowing them to adhere for 24 h, the cells were fixed with 4% PFA for 15 min. Following fixation, the cells were permeabilized and blocked by incubation in PBS containing 0.3% Triton X‐100 and 5% bovine serum albumin for 1 h. The cells were then incubated overnight at 4°C with rabbit anti‐KIF9 and mouse anti‐LAMP1 antibodies (1:1000, 1D4B‐C, DSHB, USA). On the following day, the cells were stained with DAPI (1:1000), Alexa Fluor 488‐conjugated anti‐rabbit IgG (1:1000), and Alexa Fluor 647‐conjugated anti‐mouse IgG (1:1000) for 1 h at room temperature. Images were captured using a confocal microscope (Nikon, Tokyo, Japan).

### 2.7 Co‐Immunoprecipitation (Co‐IP)

Cell lysates were prepared by adding Western and IP lysis buffer (p0013, Beyotime, Shanghai, China) with protease inhibitors (#4693116001, Roche, Basel, Switzerland). The cell suspensions were collected into sterilized 1.5 mL EP tubes and incubated on ice for 30 min. The supernatant was obtained by centrifugation at 12,000 g at 4°C for 15 min. To deplete non‐specific proteins, protein A/G magnetic beads for IP (B23202, Bimake, Shanghai, China) were added and incubated at 4°C for 30 min. The beads were removed using a magnetic rack, and protein concentration was determined using the BCA Protein Assay Kit (#23227, Thermo Scientist, MA, USA). Then, 500 μg of protein samples were incubated overnight at 4°C with either the target protein antibody or the control non‐specific IgG antibody. Magnetic beads were added to the mixture and incubated for an additional 2 h. The mixture was washed five times with pre‐cooled phosphate buffer solution (PBS) and then mixed with 5× SDS‐PAGE sample buffer. The samples were boiled at 95°C for 5 min to elute the bound proteins from the magnetic beads, and immunoblotting analysis was subsequently performed.

### 2.8 Western Blot

Cells or mouse hippocampal tissue homogenates were lysed on ice for 30 min using RIPA lysis buffer (P0013B, Beyotime, Shanghai, China) with protease and phosphatase inhibitors (#4906837001, Roche, Basel, Switzerland). The samples were centrifuged at 12,000 rpm for 15 min at 4°C, and the supernatant containing total protein was collected. Total protein concentration was determined using the BCA assay. 30 μg of the protein was mixed with 5× sample buffer and denatured by boiling at 95°C for 5 min. The samples were separated by SDS‐PAGE and transferred onto immobilon‐PTM polyvinylidene difluoride (PVDF) membranes (Millipore, MA, USA). The membranes were blocked in 10% bovine serum albumin (Sigma, St. Louis, MO, USA) at room temperature for 1 h and then incubated overnight at 4°C with primary antibodies. Following this, membranes were incubated at room temperature for 1 h with the corresponding HRP‐labeled goat anti‐rabbit IgG (1:3000; Perkin‐Elmer) or goat anti‐mouse IgG (1:3000; Perkin‐Elmer). Western ECL substrate (Bio‐Rad, Hercules, CA, USA) was applied to the membranes, which were then imaged using a Bio‐Rad Imager. Relative protein expression was quantified using Quantity One software (Bio‐Rad, Hercules, CA, USA) and normalized to GAPDH.

### 2.9 Adeno‐Associated Virus (AAV) and Microinjection

To overexpress KIF9 in vivo, mice at 2 months of age were anesthetized with an intraperitoneal injection of sodium phenobarbital (60 mg/kg) and positioned on a stereotaxic injection device. Bilateral craniotomies were performed to access the CA1 region of the hippocampus (−2.5 mm posterior and ±2.0 mm lateral to the anterior fontanel at a depth of −2.5 mm). Subsequently, 1 μL of AAV_KIF9_ (1.23 × 10^13^ TU/mL, OBiO Technology, Shanghai, China) was injected into each site. AAV_KIF9_ successfully overexpressed KIF9 in neurons, microglia, and astrocytes within the CA1, DG, and CA3 regions of the hippocampus (Figure S1). Behavioral tests were conducted when the mice reached 5 months of age.

### 2.10 Open Field Test

The open field test is used to assess motor function and anxiety‐related behavior. Mice were placed in a 40 × 40 × 60 cm open field box, equipped with an overhead high‐definition camera and surrounded by blue curtains to create a relatively isolated environment. After a 24‐h acclimation period, mice were placed in the center of the open field and allowed to explore freely for 10 min. This test was conducted during the light cycle. The entire session was recorded and analyzed using Any‐maze software (Stoelting, Wood Dale, USA).

### 2.11 Morris Water Maze Test

The Morris water maze test was used to evaluate the spatial learning abilities of the experimental animals. The test was conducted in a circular stainless steel pool with a diameter of 150 cm and a height of 50 cm. Opaque water, prepared by non‐toxic white pigment, was maintained water temperature at 24°C ± 1°C. The pool was enclosed by a blue curtain, onto which geometric shapes of different colors were affixed to serve as visual cues. A high‐definition camera was mounted directly above the pool to record the mice's activity. During the adaptation phase, mice were allowed to swim freely in the pool for 120 s. Following a 24‐h interval, the training phase commenced, during which mice were trained to locate a hidden platform (13 cm in diameter) from various starting positions. The training consisted of four trials per day for 5 consecutive days. If a mouse located the platform within 2 min and remained there for more than 3 s, the trial was considered successful. If the platform was not found within 2 min, the mouse was guided to it and allowed to remain on it for 20 s. An exploratory test phase was performed 24 h after the final training session, during which the platform was removed and mice were allowed to swim freely for 2 min. The entire session was conducted during the light cycle. The ANY‐maze tracking system was used to record and analyze the results.

### 2.12 Barnes Maze Test

The Barnes maze test was used to assess the spatial learning and memory. The experiment was conducted on a white circular platform with a diameter of 75 cm, featuring 18 evenly spaced circular holes (5 cm in diameter) around the perimeter. The platform was positioned 1 m above the ground and enclosed by a blue curtain with three distinct geometric shapes as visual cues. A high‐definition camera was placed directly above the platform to capture the mice's movements. Prior to the experiment, mice were acclimated in the chamber for 24 h. On the first day of the adaptation phase, mice were placed in the center of the platform and allowed to explore for 3 min. The training phase began the following day. One of the holes was fitted with a white opaque escape box, which could be pulled out as an escape option. Mice underwent two rounds of training per day for 5 consecutive days. If a mouse entered the escape box within 3 min, the trial ended. If the escape box was not found within this time frame, the mouse was guided to it and kept inside for 60 s. Twenty‐four hours after the final training session, the escape box was removed, and a final test was conducted where mice explored the platform for 3 min. The entire test was carried out during the light cycle. The movements were recorded and analyzed. The ANY‐maze tracking system was used to record and analyze the results.

### 2.13 Immunohistochemistry

For immunohistochemistry, mice were deeply anesthetized with an intraperitoneal injection of 1.5 g/kg urethane. Following anesthesia, PBS was perfused into the left ventricle until the limbs, tongue, and liver turned white. One hemisphere of the brain was rapidly frozen in liquid nitrogen for protein extraction, while the other hemisphere was fixed in PFA for 48 h and then dehydrated in 30% sucrose for 2 days. The brain was embedded in optimal cutting temperature (OCT) compound at −20°C, and serial coronal sections (30 μm thick) were prepared. Then, the sections were treated with 88% formic acid for 15–30 min, followed by incubated in 3% hydrogen peroxide (H_2_O_2_) for 30 min, and blocked with 10% bovine serum albumin for 1 h. The sections were then incubated overnight at 4°C with the 4G8 antibody (1:500, BioLegend). Plaques were visualized using the ABC and DAB methods, and all strained slides were scanned with a whole‐slide scanner (Olympus, Tokyo, Japan). Plaque quantification was performed using ImageJ software.

### 2.14 Statistics

Data were expressed as mean ± SEM. Statistical analysis was performed using analysis of variance (ANOVA) or two‐tailed Student's *t*‐test, as appropriate. Statistical significance was determined at *p < 0.05, **p < 0.01, and ***p < 0.001.

## 3 Results

### 3.1 KIF9 Expression Decreases in an Age‐Dependent Manner in the Hippocampus of AD Model Mice, Accompanied by Dysfunction of Macroautophagy

**FIGURE 1:**
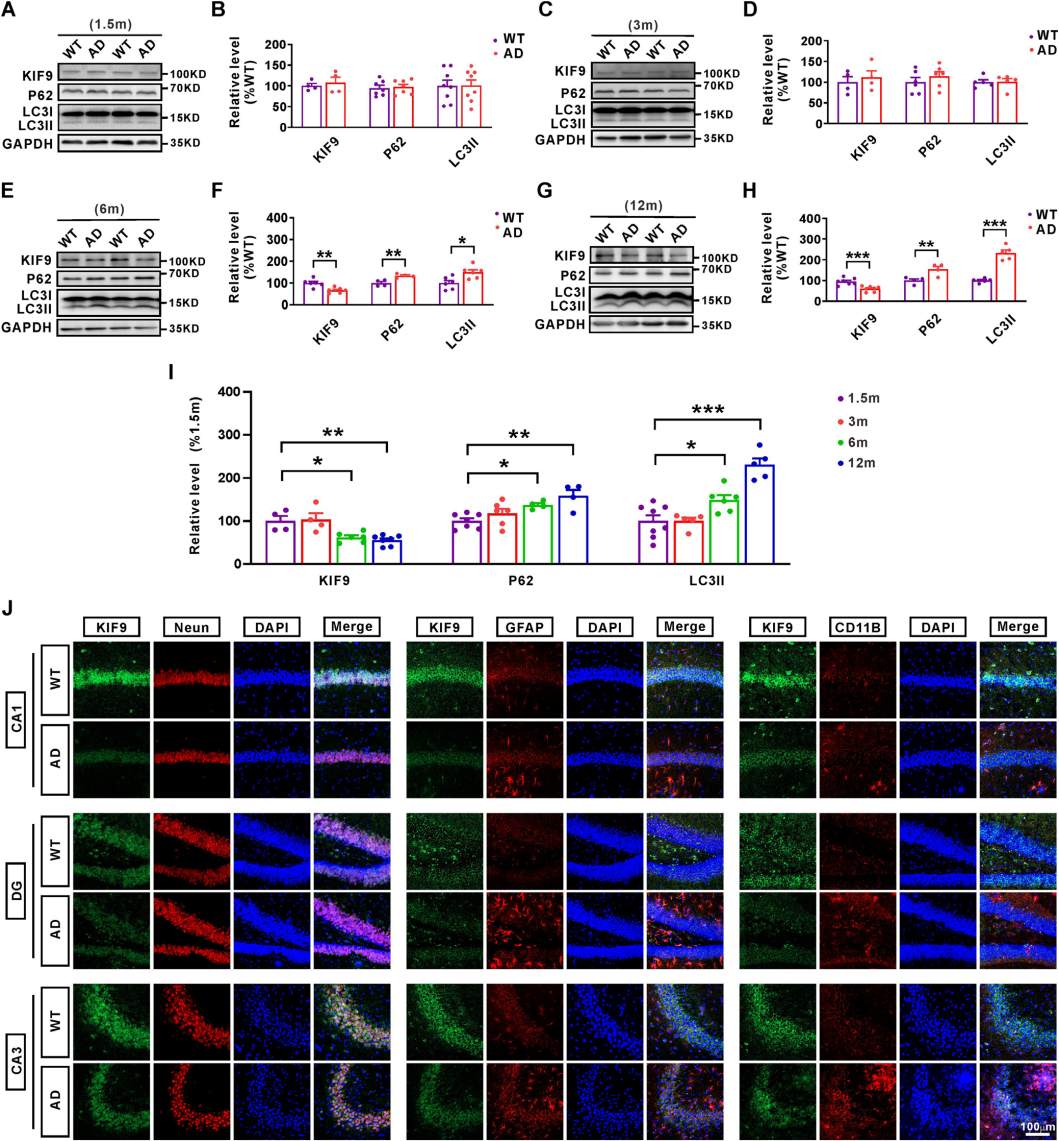
KIF9 expression decreases in an age‐dependent manner in the hippocampus of AD model mice, accompanied by dysfunction of the macroautophagy. (A–H) Western blot analysis was used to detect the protein expression levels of KIF9, P62, and LC3II in the hippocampus of wild type (WT) and APP23/PS45 double‐transgenic (AD) mice at 1.5, 3, 6, and 12 months of age. *n* = 4–8 in each group. *p < 0.05, **p < 0.01, and ***p < 0.001, as determined by unpaired Student's *t*‐test. (I) Graphical representation of changes in KIF9, P62, and LC3II protein levels with increasing age. *p < 0.05, **p < 0.01, and ***p < 0.001 by one‐way ANOVA. (J) Immunofluorescence assay was employed to assess the protein expression levels of KIF9 in neurons, astrocytes, and microglia in CA1, DG, and CA3 regions within the hippocampus of WT and AD mice. Scale bar: 100 μm. *n* = 3 in each group.

To investigate alterations in KIF9 protein expression and macroautophagy‐related proteins in AD, we collected hippocampal tissues from APP23/PS45 double transgenic mice at four different age stages (Figure 1). At 1.5 and 3 months of age, there were no significant differences in the protein levels of KIF9, p62, and LC3II between AD model mice and wild‐type (WT) mice (Figure 1A–D). However, by 6 months of age, KIF9 protein expression significantly decreased, while the protein levels of p62 and LC3II markedly increased in the hippocampus of AD model mice compared with WT mice (Figure 1E,F). In 12‐month‐old model mice, KIF9 protein levels were further reduced, with continued increases in the protein levels of p62 and LC3II (Figure 1G,H). These findings suggest that the age‐related reduction in KIF9 expression in AD model mice may be associated with the dysfunction of macroautophagy (Figure 1I). The immunofluorescent imaging assay also showed that the protein level of KIF9 was significantly decreased in neurons, microglia, and astrocytes within the hippocampus of 6‐month‐old AD model mice (Figure 1J).

### 3.2 Overexpression of KIF9 Enhances Degradation of APP Amyloidogenic Pathway Proteins via Macroautophagy

**FIGURE 2:**
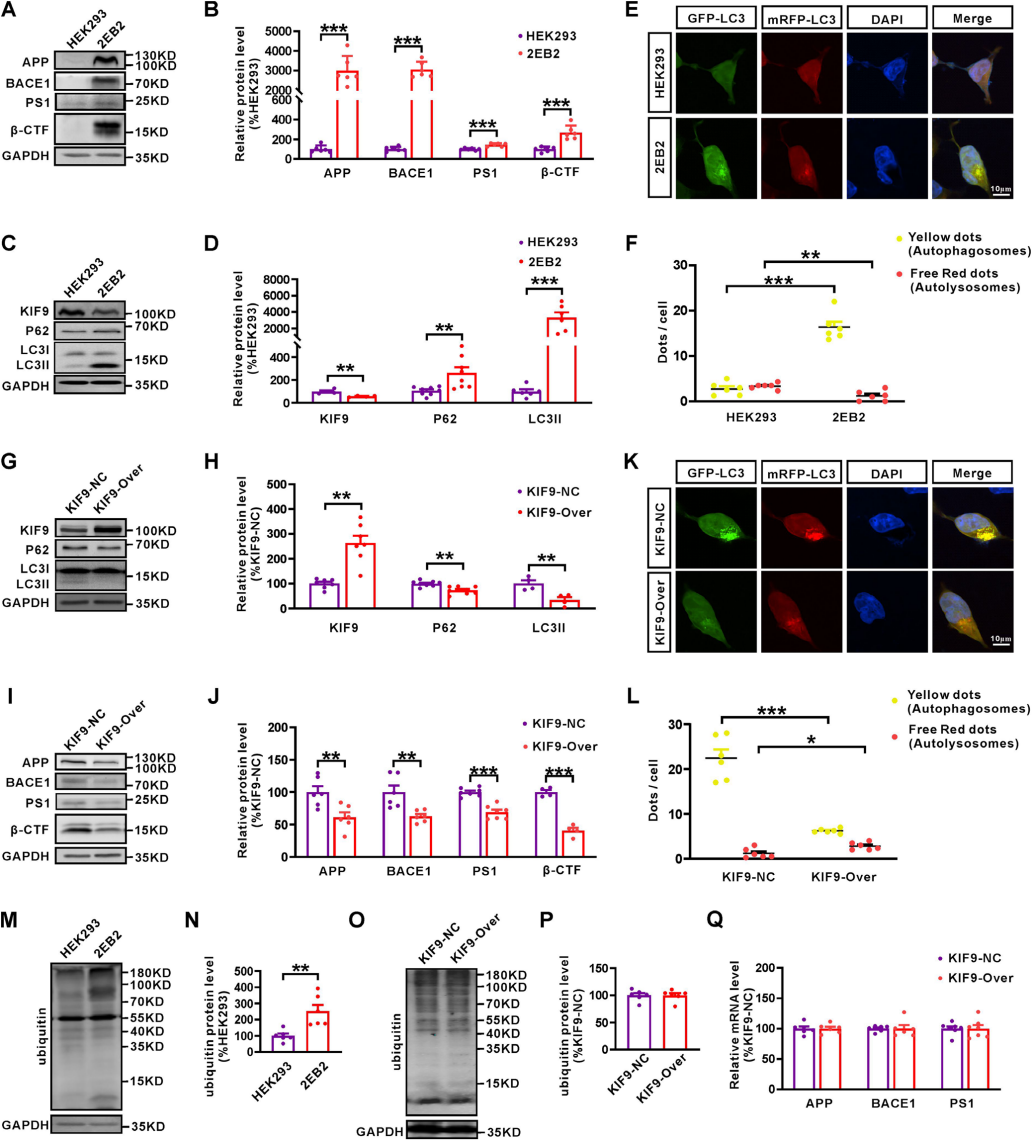
Overexpression of KIF9 promotes the degradation of APP amyloidogenic pathway‐related proteins through the macroautophagy pathway. (A, B) Western blot analysis of the protein expression levels of APP, BACE1, PS1, and β‐CTF in HEK293 and 2EB2 cells. *n* = 6 in each group. (C, D) Western blot analysis of the protein expression levels of KIF9, P62, and LC3II in HEK293 and 2EB2 cells. *n* = 4–8 in each group. (E, F) Confocal microscopy was used to detect fluorescent signals of GFP‐mRFP‐LC3. The analysis was conducted in six randomly selected fields, with 1–4 cells randomly chosen from each field. Scale bar: 10 μm. (G, H) Western blot analysis of the protein expression levels of KIF9, P62, and LC3II in 2EB2 cells transfected with the KIF9 overexpression plasmid (KIF9‐OVER) or its control plasmid (KIF9‐NC). *n* = 4–8 in each group. (I, J) Western blot analysis of the protein expression levels of APP, BACE1, PS1, and β‐CTF in KIF9‐overexpressing versus control cells. *n* = 4–7 in each group. (K, L) Confocal microscopy was used to detect fluorescence signals of GFP‐mRFP‐LC3. The analysis was conducted in six randomly selected fields, with 1–4 cells randomly chosen from each field. Scale bar: 10 μm. (M, N) Western blot analysis of ubiquitin protein levels in HEK293 and 2EB2 cells. *n* = 6 in each group. (O, P) Western blot analysis of ubiquitin protein levels in KIF9‐overexpressing versus control cells. *n* = 6 in each group. (Q) RT‐qPCR analysis of APP, BACE1, and PS1 mRNA levels in 2EB2 cells transfected with the KIF9‐NC or KIF9‐overexpressing plasmid. *n* = 6–7 in each group. *p < 0.05, **p < 0.01, and ***p < 0.001 by unpaired Student's *t*‐test.

To further explore the relationship between the age‐related reduction of KIF9 and the dysfunction of macroautophagy in AD, we examined the protein levels of KIF9, p62, and LC3II in HEK293 cells stably transfected with human Swedish mutant APP695 and BACE1 (2EB2 cells). Compared to HEK293 cells, 2EB2 cells exhibited notable overexpression of APP and BACE1 (Figure 2A,B), as well as upregulation of proteins related to the APP amyloidogenic pathway, such as PS1 and β‐CTF (Figure 2A,B). In 2EB2 cells, KIF9 protein levels were significantly reduced (Figure 2C,D), and there was a significant increase in the protein levels of p62 and LC3II. HEK293 and 2EB2 cells were transfected with the GFP‐mRFP‐LC3 plasmid for 24 h (Figure 2E,F). Co‐localization of GFP and mRFP was observed. Yellow dots indicate autophagosomes, while free red dots represent autolysosomes. Compared to HEK293 cells, 2EB2 cells showed a significant increase in autophagosomes (yellow dots) and a marked reduction in autolysosomes (free red dots). These results align with observations from AD animal models aged 6 months and older, suggesting a potential link between reduced KIF9 expression and the dysfunction of macroautophagy in AD.

To investigate the role of KIF9 in AD pathology, we overexpressed KIF9 in 2EB2 cells. Compared to the negative control group (KIF9‐NC), KIF9 overexpression significantly reduced the protein levels of p62 and LC3II (Figure 2G,H) but had no notable effect on cell viability (Figure S2A). Additionally, overexpression of KIF9 led to a significant reduction in APP protein levels and proteins associated with the amyloidogenic pathway, including BACE1, PS1, and β‐CTF (Figure 2I,J). To investigate the effect of KIF9 on autophagic flux in 2EB2 cells, we transfected the cells with either the KIF9 overexpression (KIF9‐Over) or its control (KIF9‐NC) plasmid for 4 h, followed by transfection with the GFP‐mRFP‐LC3 plasmid for 24 h. The results showed that KIF9 overexpression significantly reduced the number of autophagosomes (yellow dots) while increasing the number of autolysosomes (free red dots) in 2EB2 cells (Figure 2K,L). These results suggest that KIF9 overexpression alleviates macroautophagy pathway dysfunction and enhances the degradation of APP and amyloidogenic pathway proteins by promoting autophagic flux.

Given that proteins are primarily degraded via the ubiquitin‐proteasome system and/or the autophagolysosome system, we assessed total ubiquitination levels in HEK293 and 2EB2 cells. The results showed a significant increase in total ubiquitination levels in 2EB2 cells compared to HEK293 cells (Figure 2M,N), consistent with our observations in the hippocampus of WT and AD model mice aged 6 months and older (Figure S3). However, overexpression of KIF9 in 2EB2 had no significant effect on the total ubiquitination level (Figure 2O,P). RT‐qPCR analysis of APP, BACE1, and PS1 transcription levels after KIF9 overexpression revealed no significant changes, indicating that KIF9's effects are likely post‐transcriptional (Figure 2Q). These findings support the hypothesis that KIF9 facilitates the degradation of APP amyloidogenic pathway‐related proteins through enhanced macroautophagy.

### 3.3 KIF9 Promotes Macroautophagy by Mediating Lysosomal Transport

**FIGURE 3:**
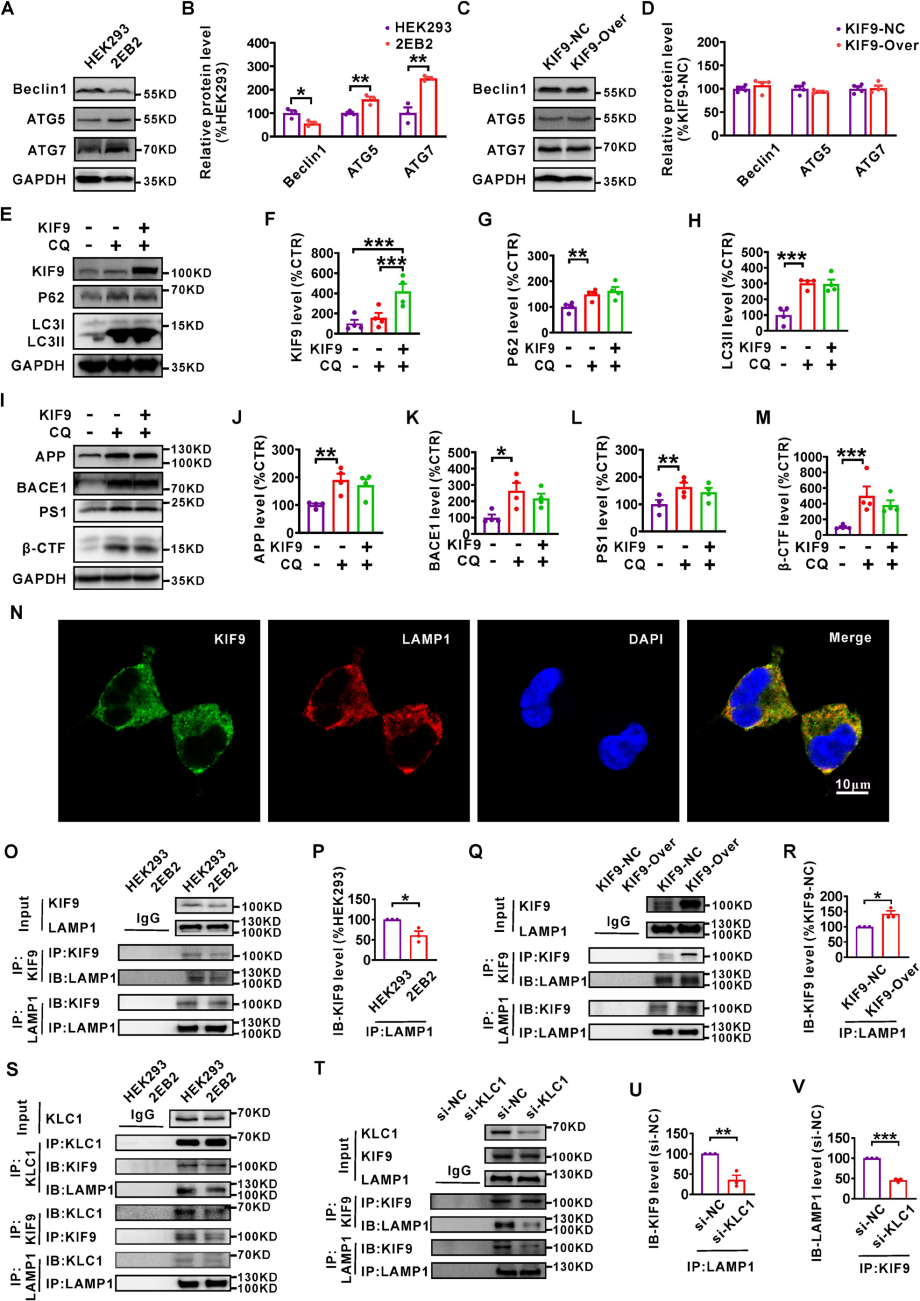
KIF9 promotes macroautophagy by mediating lysosomal transport. (A, B) Western blot analysis of the protein expression levels of Beclin1, ATG5, and ATG7 in HEK293 and 2EB2 cells. *n* = 3 in each group. (C, D) Western blot analysis of the protein expression levels of Beclin1, ATG5, and ATG7 in KIF9‐overexpressing versus control cells. *n* = 4 in each group. (E–H) Western blot analysis of KIF9, P62, and LC3II proteins in cells transfected with the KIF9 overexpression plasmid, followed by co‐treatment with chloroquine (CQ, 50 μM, 24 h). *n* = 4 in each group. (I–M) Western blot analysis of APP, BACE1, PS1, and β‐CTF proteins in 2EB2 cells treated with CQ (50 μM) and transfected with KIF9 for an additional 24 h. *n* = 4 in each group. *p < 0.05, **p < 0.01, and ***p < 0.001 by one‐way ANOVA. (N) Immunofluorescence staining of KIF9 and the lysosome marker LAMP1, followed by confocal imaging. Scale bar: 10 μm. *n* = 10 randomly selected fields. (O) Co‐IP assay assessing the interactions between KIF9 and LAMP1 in HEK293 and 2EB2 cells. *n* = 3 in each group. (P) Levels of KIF9 protein interacting with LAMP1 following immunoprecipitation (IP: LAMP1) in HEK293 and 2EB2 cells. *n* = 3 in each group. *p < 0.05 by unpaired Student's *t*‐test. (Q, R) Interaction between KIF9 and LAMP1 after KIF9 overexpression in 2EB2 cells, with measurement of KIF9 protein levels obtained through LAMP1 interaction after IP: LAMP1. *n* = 3 in each group. (S) Co‐IP assays detecting endogenous interactions between KLC1, KIF9, and LAMP1 in HEK293 and 2EB2 cells. *n* = 3 in each group. (T) Effects of small interfering RNA targeting KLC1 (si‐KLC1) and negative control (si‐NC) on the endogenous interaction between KIF9 and LAMP1, evaluated through Co‐IP assays. *n* = 3 in each group. (U) Interaction between KIF9 and LAMP1 following KLC1 knockdown in 2EB2 cells, with measurement of KIF9 protein levels obtained through LAMP1 interaction after IP: LAMP1. *n* = 3 in each group. **p < 0.001 by unpaired Student's *t*‐test. (V) Interaction between KIF9 and LAMP1, and LAMP1 protein levels obtained through KIF9 interaction following KLC1 knockdown in 2EB2 cells, evaluated by Co‐IP. *n* = 3 in each group. ***p < 0.001 by unpaired Student's *t*‐test.

Given that the increase in P62 and LC3II protein levels was primarily due to increased autophagosome formation and/or decreased lysosomal degradation, we evaluated proteins associated with autophagosome formation (Beclin1, AT5, and ATG7) in HEK293 and 2EB2 cells. The results showed that, compared to HEK293 cells, Beclin1 protein levels were significantly downregulated in 2EB2 cells, while ATG5 and ATG7 protein levels were significantly increased. However, KIF9 overexpression had no significant effect on these levels (Figure 3A–D). Meanwhile, lysosomal degradation capability was assessed using the DQ‐Red BSA assay. The results showed that lysosomal degradation capability was markedly reduced in 2EB2 cells compared to HEK293 cells, while KIF9 overexpression did not have a significant effect on lysosomal degradation capability (Figure S4). These results suggest that KIF9 does not affect the process of autophagosome formation or lysosomal degradation capability.

To further investigate the mechanism by which KIF9 promotes macroautophagy in AD, we transfected the KIF9 plasmid into 2EB2 cells for 4 h, followed by co‐treatment with the macroautophagy inhibitor CQ (50 μM) for an additional 24 h. Notably, all these treatments did not affect cell viability (Figure S2B). The results showed that although KIF9 protein levels in the CQ group exhibited an increase compared to the untreated group, this difference was not statistically significant. However, KIF9 protein expression was significantly elevated in 2EB2 cells co‐treated with the KIF9 plasmid and CQ (KIF9 + CQ; Figure 3E,F). Notably, no significant differences in the protein levels of P62 and LC3II were observed between 2EB2 cells co‐treated with the KIF9 plasmid and CQ or CQ alone (Figure 3E,G,H). These results suggest that KIF9 does not reverse the inhibitory effect of CQ on lysosome–autophagosome fusion. In addition, the protein levels of APP, BACE1, PS1, and β‐CTF significantly increased in the CQ group compared to the control group. However, these levels did not show significant changes in the KIF9 + CQ group compared to the CQ group (Figure 3I–M), indicating that KIF9 can promote the degradation of APP amyloidogenic pathway‐related proteins through the autophagy–lysosome pathway.

Next, to determine whether KIF9 increases the occurrence of macroautophagy by promoting lysosome transport, we performed immunofluorescence staining and Co‐IP assays. Immunofluorescence staining revealed substantial colocalization of KIF9 with the lysosome marker LAMP1 in 2EB2 cells (Figure 3N). Co‐IP assays further demonstrated a significant interaction between KIF9 and LAMP1 in both HEK293 and 2EB2 cells (Figure 3O). In 2EB2 cells, the level of the KIF9 protein obtained from LAMP1 interaction was significantly reduced compared to HEK293 cells (Figure 3P), likely due to lower KIF9 levels in 2EB2 cells. After KIF9 overexpression, the amount of KIF9 obtained from LAMP1 interaction significantly increased (Figure 3Q,R), suggesting that KIF9 overexpression enhances its interaction with lysosomes.

**FIGURE 4:**
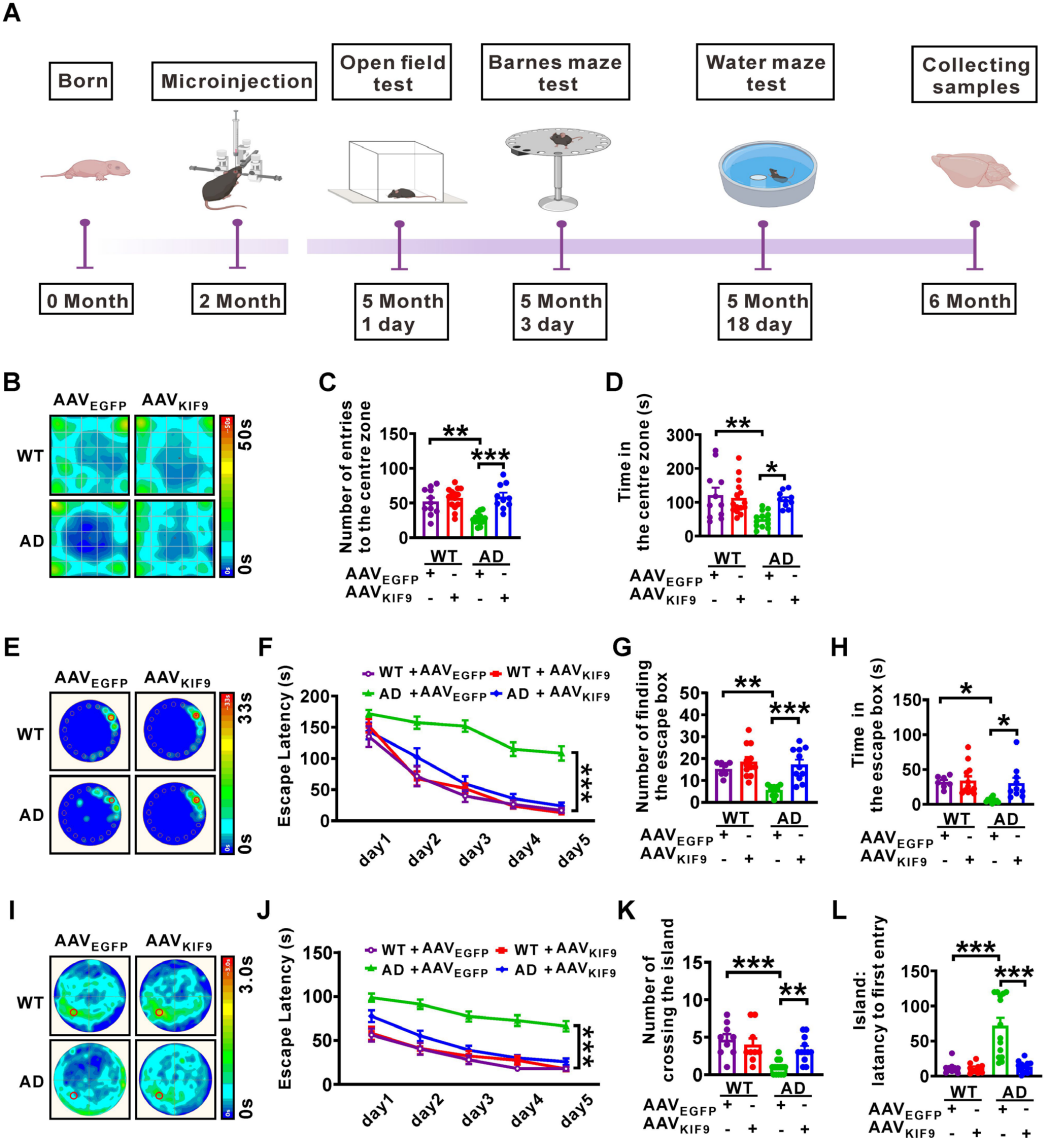
Overexpression of KIF9 ameliorates cognitive dysfunction in AD model mice. (A) Experimental design diagram illustrating the animal study. (B) Average heatmap of mouse activity during the open field test. (C) Number of entries into the center zone. (D) Time spent in the center zone. *n* = 10–15 in each group. *p < 0.05, **p < 0.01, and ***p < 0.001 by one‐way ANOVA. (E) Average heatmap during memory retrieval in the Barnes maze test. (F) Latency to the escape box during spatial learning in the Barnes maze paradigm. *n* = 7–12 in each group. ***p < 0.001 by repeated measures ANOVA. (G) Number of times the escape box was found during memory retrieval. (H) Time spent in the escape box during memory retrieval. *n* = 7–12 in each group. *p < 0.05, **p < 0.01, and ***p < 0.001 by one‐way ANOVA. (I) Average heatmap during memory retrieval in the Morris water maze test. (J) Escape latency to find the hidden platform during spatial learning in the Morris water maze. *n* = 9–14 in each group. ***p < 0.001 by repeated measures ANOVA. (K) Number of entries into the platform zone during memory retrieval. (L) Latency to the first entry into the platform zone during memory retrieval. *n* = 9–14 in each group. **p < 0.01 and ***p < 0.001 by one‐way ANOVA.

Given that specific kinesins can connect lysosomes via their light chain 1 (KLC1) and facilitate their transport along microtubules (Farías et al. 2017; Guardia et al. 2016; Pu et al. 2016; Rosa‐Ferreira and Munro 2011), we examined the potential involvement of KIF9 in lysosomal transport. The Co‐IP results showed that KLC1 interacted significantly with both KIF9 and the lysosome marker LAMP1 (Figure 3S). To explore the role of KLC1 in mediating the KIF9–lysosome association, we used small interfering RNA (si‐KLC1) to knock down KLC1 in HEK293 cells (Figure 4T). KLC1 knockdown did not affect the expression of KIF9 or LAMP1, nor did it impact cell viability compared to the control group (si‐NC). However, it significantly reduced the interaction between KIF9 and LAMP1 (Figure 4T–V and Figure S2C). These results suggest that reduced KIF9 expression in AD impairs its lysosomal transport function via KLC1. Notably, overexpression of KIF9 failed to restore the reduced interaction between KIF9 and LAMP1 caused by KLC1 knockdown (Figure S5) and did not affect cell activity (Figure S2D). Thus, the lysosomal degradation of APP amyloidogenic pathway‐related proteins is likely associated with KIF9‐mediated lysosomal transport through KLC1.

### 3.4 Overexpression of KIF9 Ameliorates Cognitive Dysfunction in AD Model Mice

Since overexpression of KIF9 reduced APP amyloidogenic processing in vitro, we next wanted to evaluate the potential therapeutic effect of KIF9 overexpression on cognitive function. We used AAV vectors carrying KIF9 (AAV_KIF9_) to deliver KIF9 to the hippocampus of APP23/PS45 double transgenic mice. Mice were bilaterally microinjected with AAV_KIF9_ or its control (AAV_EGFP_) at 2 months of age. Behavioral testing was performed at 5 months, and brain tissue samples were collected for pathological analysis at 6 months (Figure 4A).

To assess anxiety‐like behavior, we conducted an open field test (Figure 4B–D). In comparison with WT + AAV_EGFP_ controls, there were no significant differences in the number of entries to the center zone or the time in the center zone for the WT + AAV_KIF9_ group. However, AD + AAV_EGFP_ mice showed a significant reduction in both the number of entries to the center zone and the time spent there. In contrast, AD + AAV_KIF9_ group exhibited a significant increase in these measures compared to the AD + AAV_EGFP_ group. These results suggest that KIF9 overexpression can ameliorate anxiety‐like behavior in AD model mice.

We further evaluated the effect of KIF9 overexpression on hippocampal‐dependent learning and memory in AD model mice using the Barnes maze and Morris water maze tests. In the Barnes maze test (Figure 4E–H), AD + AAV_EGFP_ mice exhibited significant impairment in spatial learning, as indicated by longer escape latencies to find the escape box compared to WT + AAV_EGFP_ mice. The WT + AAV_KIF9_ group showed no significant differences from WT + AAV_EGFP_ controls. Importantly, KIF9 overexpression (AD + AAV_KIF9_) significantly shortened the escape latency in AD model mice. During the probe trial, AD + AAV_EGFP_ mice displayed impaired spatial memory retrieval, characterized by fewer entries into the escape box and reduced time spent there compared to WT + AAV_EGFP_ mice. Conversely, KIF9 overexpression restored memory retrieval in AD model mice to levels comparable to WT controls.

Similarly, the Morris water maze test (Figure 4I–L) revealed significant spatial learning deficits in AD + AAV_EGFP_ mice, which took longer to reach the hidden platform compared to WT + AAV_EGFP_ controls. Overexpression of KIF9 significantly reduced the time required for AD model mice to locate the hidden platform. No significant differences were observed between WT + AAV_KIF9_ and WT + AAV_EGFP_ groups. In the probe trial, AD + AAV_EGFP_ mice showed impaired spatial memory retrieval, indicated by fewer platform crossings and increased latency to the first entry into the platform zone compared to WT + AAV_EGFP_ mice. However, KIF9 overexpression restored these measures to normal levels.

### 3.5 Overexpression of KIF9 Reduces Macroautophagy‐Related Proteins and Amyloid Pathology in AD Model Mice

**FIGURE 5:**
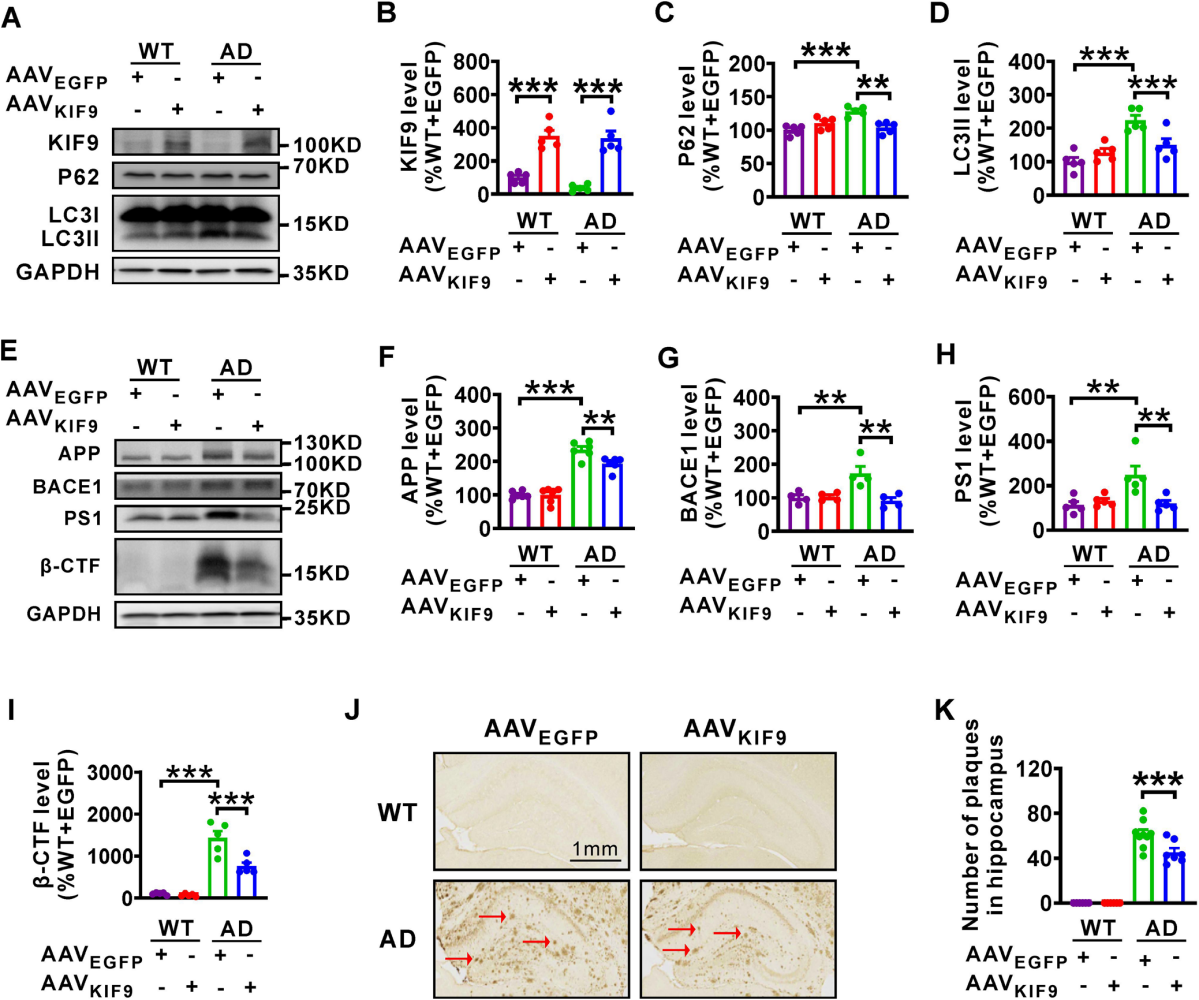
Overexpression of KIF9 reduces the expression of macroautophagy‐related proteins and ameliorates pathology in AD model mice. (A–D) Western blot analysis of KIF9, P62, and LC3II in hippocampal tissue from mice subjected to various treatments. *n* = 5 in each group. **p < 0.01 and ***p < 0.001 by one‐way ANOVA. (E–I) Western blot analysis of APP, BACE1, PS1, and β‐CTF proteins in hippocampal tissue from mice subjected to various treatments. *n* = 4–6 in each group. **p < 0.01 and ***p < 0.001 by one‐way ANOVA. (J, K) Number of senile plaques in the hippocampus of WT and AD model mice receiving AAV_EGFP_ or AAV_KIF9_ microinjection. Scale bars = 1 mm. *n* = 48 to 72 slices from 6 to 9 mice in each group. ***p < 0.001 by one‐way ANOVA.

To investigate whether KIF9 overexpression could alleviate autophagosome accumulation, we assessed levels of P62 and LC3II (Figure 5A–D). Compared to WT + AAV_EGFP_ controls, these markers were significantly elevated in AD + AAV_EGFP_ mice, consistent with our in vitro findings. Importantly, AAV_KIF9_ treatment restored P62 and LC3II protein levels in AD model mice.

We also evaluated the impact of KIF9 overexpression on amyloid pathology by measuring protein levels of APP, BACE1, PS1, and β‐CTF (Figure 5E–I). No significant differences were observed between WT + AAV_EGFP_ and WT + AAV_KIF9_ groups. However, AD + AAV_EGFP_ mice had significantly elevated levels of these proteins. KIF9 overexpression (AD + AAV_KIF9_) significantly reduced the levels of APP, BACE1, PS1, and β‐CTF in AD model mice, though not to the levels seen in WT mice (AD + AAV_EGFP_). Since the deposition of Aβ peptides, which leads to the formation of senile plaques, is a hallmark of AD, we examined the formation of senile plaque in the hippocampus (Figure 5J,K). There was no significant difference in plaque number between WT + AAV_EGFP_ and WT + AAV_KIF9_ groups. AD + AAV_EGFP_ mice showed a significant increase in hippocampal senile plaques compared to WT + AAV_EGFP_ controls. Notably, KIF9 overexpression through AAV_KIF9_ significantly reduced the number of senile plaques in AD model mice.

**FIGURE 6:**
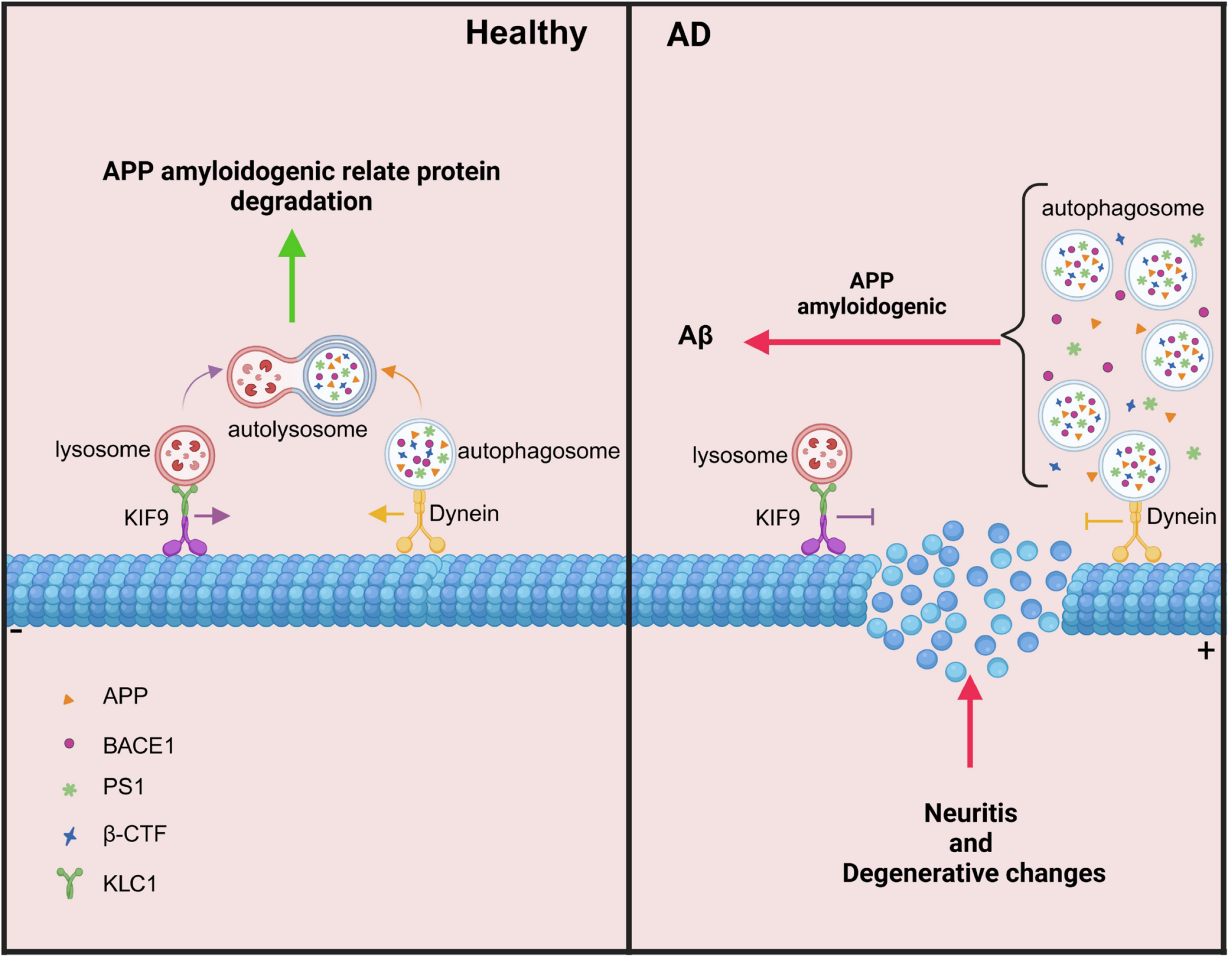
Schematic illustration postulated the mechanism of KIF9 in AD. KIF9 mediates lysosomal anterograde transport via its light chain KLC1, promoting the degradation of APP amyloid pathway‐related proteins in AD through the macrophage pathway. (Created with BioRender.com).

## 4 Discussion

Alzheimer's disease is a neurodegenerative disorder prevalent among the elderly, characterized by abnormal protein aggregation and accumulation in the brain. Macroautophagy, an essential pathway for lysosomal degradation of cellular cargo, plays a pivotal role in clearing these misfolded proteins (Chandran, Oliver, and Rochet 2023; Jiang, Kuo, and Arkin 2023; Liu et al. 2024). Dysregulation of macroautophagy has been implicated in neurodegenerative diseases such as AD (Longobardi et al. 2024). Our findings align with previous studies, demonstrating progressive impairment of autophagy in the hippocampus of AD model mice with age, resulting in compromised fusion between autophagosomes and lysosomes. This impairment is also observed in AD model cells.

Recent study has shown that abnormal vesicle accumulation in dystrophic neurites within the brains of AD patients (Suzuki and Terry 1967), which were later characterized as autophagosomes (Nixon et al. 2005). The abnormal accumulation of autophagosomes results from impaired retrograde transport of autophagosomes mediated by dyneins and anterograde transport of lysosomes mediated by kinesins (Long et al. 2020). This impairment disrupts the fusion of autophagosomes and lysosomes, exacerbating AD progression (Sanchez‐Varo et al. 2012). Recent research has shown that upregulating dynein intermediate chain (DIC) promotes retrograde axonal transport of autophagosomes, restores autophagy, and improves Aβ clearance and cognitive function in APP/PS1 double‐transgenic mice (Zhou et al. 2020). Similarly, curcumin, a polyphenol plant compound, was found to enhance dynein‐mediated retrograde autophagy along axons by upregulating the expression of DIC and scaffold proteins in N2A/APP695swe cells (an AD model cell stably transfected with APP695swe in N2A cells), thereby promoting autophagy (Liang et al. 2019). Furthermore, another study has demonstrated that overexpression of KIF5A enhances anterograde transport of lysosomes, reversing the neurotoxic inhibition of macroautophagy induced by trimethyltin chloride (Liu et al. 2021). While KIF9, a member of the kinesin superfamily, also mediates intracellular cargo transport similar to KIF5A, its role in autophagy and AD has not been extensively reported. We propose that KIF9 facilitates the anterograde transport of lysosomes, promoting the fusion of autophagosomes and lysosomes, thereby helping to attenuate the progression of AD. Indeed, KIF9 expression is significantly reduced in the hippocampus of AD model mice and in AD model cells. Overexpression of KIF9 not only promotes the degradation of proteins associated with the amyloidogenic pathway of APP through macroautophagy but also improves cognitive function, autophagosome–lysosome fusion, and pathological features in AD model mice.

Kinesins are crucial for the normal transport of molecules and organelles along microtubules. They possess highly conserved structures, with all kinesins attaching to microtubules via the motor domain of their heavy chain (Nitta et al. 2004) and interacting with specific cargo through the tail domain of their light chains (Miki, Okada, and Hirokawa 2005), thereby mediating intracellular cargo transport. Most kinesins belong to the N‐kinesin subtype, which primarily facilitates anterograde transport of cargo (Kollmar and Glöckner 2003). Lysosomes, essential for cellular degradation and recycling, are primarily located in the perinuclear region (Jongsma et al. 2016). During macroautophagy, kinesin‐mediated anterograde transport of lysosomes along microtubules ensures their fusion with autophagosomes. For instance, dysfunction of Kinesin‐1 impairs lysosomal anterograde transport to axons, hindering fusion and maturation with autophagosomes, and leading to diseases such as Niemann‐Pick (Yap and Winckler 2021). Recent studies have shown that various kinesins participate in lysosomal anterograde transport, including Kinesin‐1 (KIF5A, KIF5B, and KIF5C) (Farías et al. 2017), Kinesin‐2 (KIF3) (Loubéry et al. 2008), Kinesin‐3 (KIF1A and KIF1B) (Balabanian et al. 2022; Hummel and Hoogenraad 2021), and Kinesin‐13 (KIF2) (Pu et al. 2016). Our study hypothesizes that KIF9, as a member of the N‐kinesin subtype, may also mediate the anterograde transport of lysosomes along microtubules. Indeed, interaction assays revealed a significant interaction between KIF9 and the lysosomal marker LAMP1, and overexpression of KIF9 increased this interaction in 2EB2 cells.

Kinesin‐1 is the most extensively studied kinesin regarding lysosomal transport. Its heavy chains (KIF5A, KIF5B, and KIF5C) can recognize lysosomal subunits of the multi‐subunit BORC complex, Arf‐like small GTPase (Arl8), Arl8 effector proteins (such as SifA), and the kinesin‐interacting protein (SKIP) via its light chains (KLC1, KLC2, KLC3, or KLC4), mediating anterograde lysosomal transport (De Pace et al. 2024; Keren‐Kaplan and Bonifacino 2021). The expression levels of various KLCs vary among species and cell types, but all four homologous KLCs (KLC1 to KLC4) are highly conserved, with KLC1 being particularly abundant in neurons (Cabeza‐Arvelaiz et al. 1993). In the present study, we found that KLC1 acts as a connecting link in the interaction between KIF9 and lysosomes. However, while these findings provide indirect evidence for the involvement of KIF9 in anterograde lysosomal transport, direct evidence confirming this function is still needed. Further studies are needed to elucidate this potential role in the future.

In conclusion, our study suggests that KIF9 plays a crucial role in the pathogenesis of AD. Our data indicate that KIF9, through its light chain KLC1, mediates the transport of lysosomes, facilitating the degradation of APP amyloidogenic pathway‐related proteins via the macroautophagy pathway in AD. This mechanism helps reduce Aβ production in the hippocampus of AD model mice, decrease senile plaque formation, and improve cognitive function and pathological changes (Figure 6) These findings reveal a new role for KIF9 in AD and provide a scientific basis for developing KIF9‐based therapies to address learning and memory deficits in AD patients.

## Author Contributions

Zhifang Dong, Yehong Du, and Maoju Wang conceived and designed this project. Maoju Wang, Song Guo, and Lilin Yi performed biochemical assays. Maoju Wang and Zhaolun Li performed behavioral studies. Maoju Wang, Xiuyu Shi, and Yan He constructed plasmids. Maoju Wang and YePeng Fan performed immunofluorescence. Maoju Wang and Man Luo analyzed the data. Zhifang Dong, Yehong Du, and Weihong Song contributed essential reagents or tools. Maoju Wang, Yehong Du, and Zhifang Dong wrote the manuscript. All authors reviewed and edited the manuscript and approved the final version.

## Conflicts of Interest

The authors declare no conflicts of interest.

## Supporting information


Appendix S1

## Data Availability

The data that support the findings of this study are available from the corresponding author upon reasonable request.
