# Supplementary material for: KIF9 Ameliorates Neuropathology and Cognitive Dysfunction by Promoting Macroautophagy in a Mouse Model of Alzheimer's Disease
Source: Aging Cell. 2025 Jan 19;24(5):e14490. doi: 10.1111/acel.14490 (PMC12073912; doi:10.1111/acel.14490)
Supplement: Supplementary file 1 — Appendix S1 [file ACEL-24-e14490-s001.docx]

**Supplementary Materials**

**Supplementary methods**

**Immunofluorescence**

To detect the expression of KIF9 in the hippocampus of mice, the immunofluorescent assay was performed. Coronal frozen sections of brain were washed three times with PBST (PBS containing 0.05% Tween-20) for 5 min each, and then blocked with 10% bovine serum albumin at room temperature for 1h. Subsequently, the brain slices were incubated with primary antibody at 4℃ overnight. On the second day, after washing with PBST, slices were incubated with goat anti-Rat secondary antibodies conjugated to Alexa Fluor dyes and DAPI. Then slices were washed with PBST and were mounted on the slides with an antifade mounting medium. Images were acquired using a confocal microscope (Nikon, Tokyo, Japan).

**Cell viability assay**

2EB2 cells were inoculated in 96-well plates overnight at a density of 5 × 10^3^ cells/well, and then treated differently for 24 or 48 hours. Next, 10µl Cell Counting Kit-8 (CCK8, MCE, Shanghai, China) reagent was added to each well and incubated at 37℃ away from light for 2 h. The cell viability was assessed by reading absorbance at 450nm using a microplate reader (Thermo Fisher Scientific, Waltham, MA, USA).

**Lysosome degradation capability detection**

The experiment was conducted using the DQ-Red BSA kit (MedChemExpress) according to the manufacturer's protocol. Cells were seeded on coverslips in 12-well plates, and 2EB2 cells were transfected with either KIF9-NC or KIF9 overexpression plasmids. After 20 hours of transfection, the cells were incubated with a medium containing DQ-Red BSA (10 µg/ml) for 4 hours. Subsequently, the cells were washed once with 1× PBS, fixed with 4% paraformaldehyde (PFA) for 10 min, and then stained with DAPI for 20 min. After staining, the cells were washed three times with 1× PBS and immediately observed under a fluorescence microscope (Nikon, Tokyo, Japan). The fluorescence intensity of DQ-Red BSA was quantified using ImageJ software (ImageJ, NIH). Lysosomal degradation capability was expressed as the relative fluorescence intensity.

**Supplementary Figures**


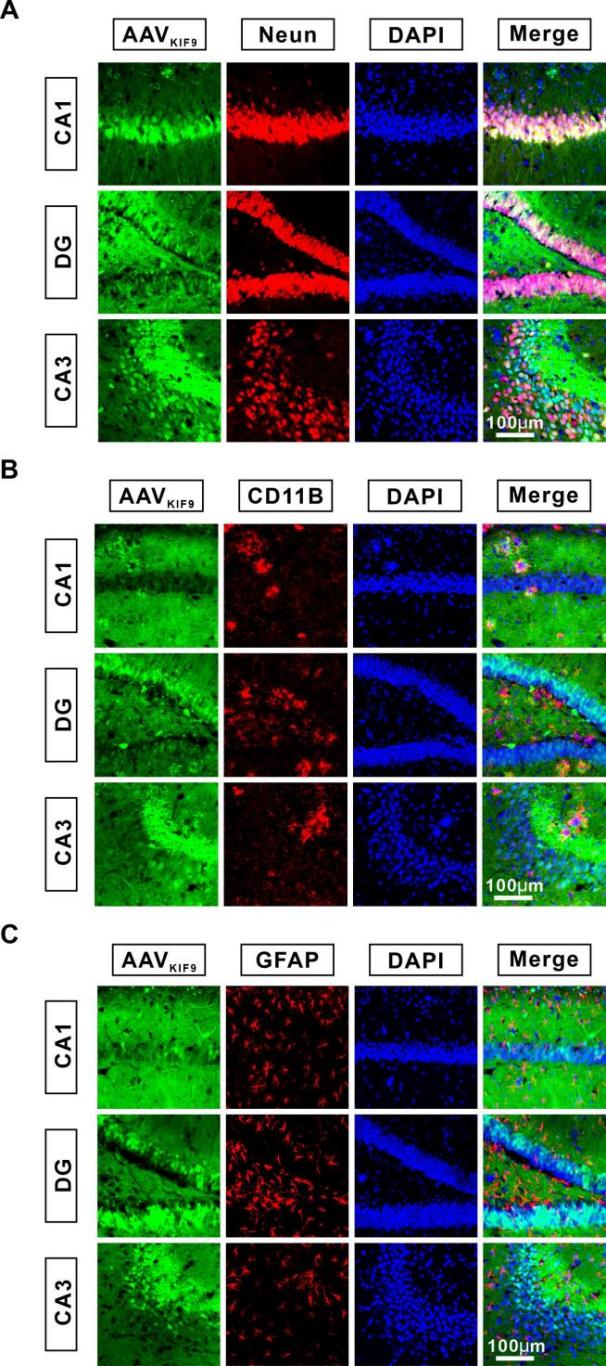


**Supplementary Fig. 1 AAV_KIF9_ successfully overexpressed KIF9 in neurons, microglia, and astrocytes in the hippocampal CA1, DG, and CA3 regions.** (A) Immunofluorescence staining of neuronal marker NueN (red) in CA1, DG, and CA3 regions of the hippocampus of APP23/PS45 mice treated with AAV_KIF9_ (green). (B) Immunofluorescence staining of microglial marker CD11B (red) in CA1, DG, and CA3 regions of AD model mice treated with AAV_KIF9_ (green). (C) Immunofluorescence staining of astrocyte marker GFAP (red) in CA1, DG, and CA3 regions of AD model mice treated with AAV_KIF9_ (green). Scale bar, 100 μm. n = 3 in each group.

**
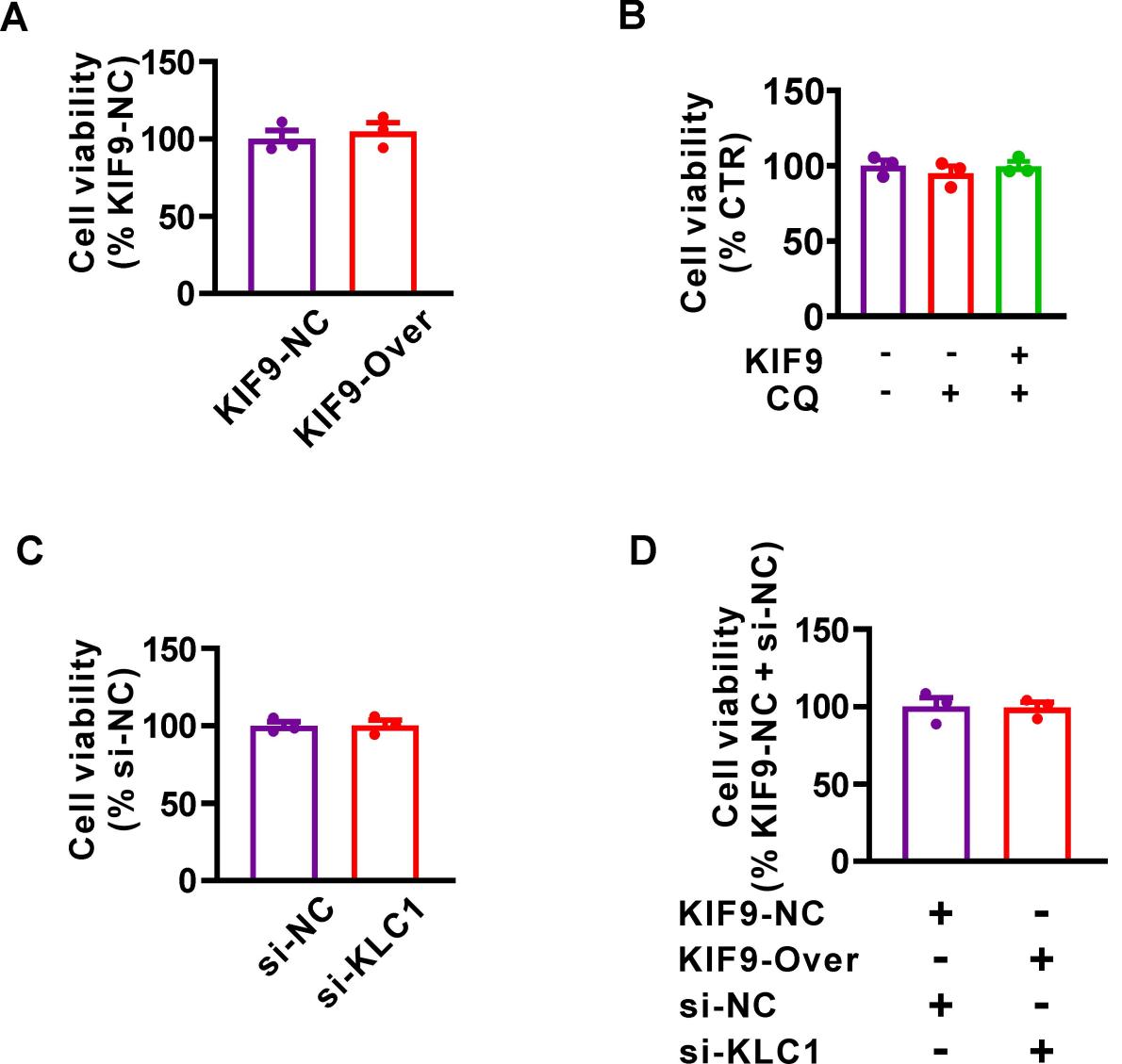
**

**Supplementary Fig. 2 All treatments had no effect on cell viability.** (A) CCK8 assay was used to detect the viability of 2EB2 cells transfected with KIF9-overexpressed plasmid (KIF9-over) or its control plasmid (KIF9-NC). n = 3 in each group. (B) CCK8 evaluated the viability of cells transfected with KIF9 overexpression plasmid and co-treatment with chloroquine (CQ, 50 μM, 24 hours). n = 3 in each group. (C) Effects of small interfering RNA targeting KLC1 (si-KLC1) and negative control (si-NC) on the cell viability was evaluated by CCK8 analysis. n = 3 in each group. (D) CCK8 assay was employed to evaluate the impact of KLC1 knockdown while overexpressing KIF9 on cell viability. n = 3 in each group.


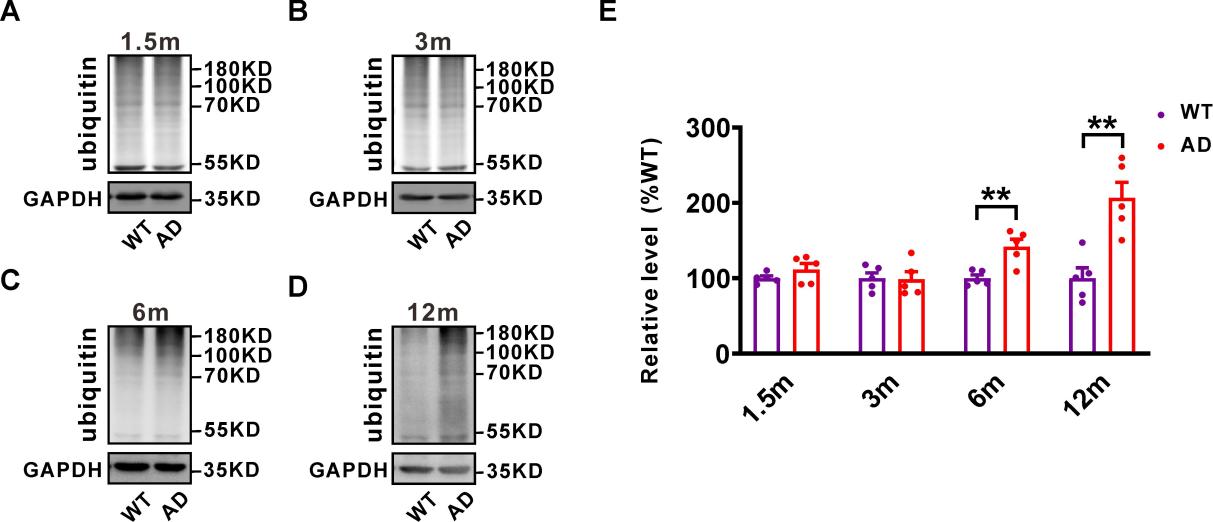


**Supplementary Fig. 3 The level of ubiquitination increases in an age-dependent manner in the hippocampus of AD model mice.** (A-E) Western blot analysis was employed to evaluate the protein expression level of ubiquitin in the hippocampus of wild type (WT) and APP23/PS45 double transgenic (AD) mice at 1.5, 3, 6 and 12 months of age. n=5 in each group. **p<0.001 by unpaired Student’s t-test.


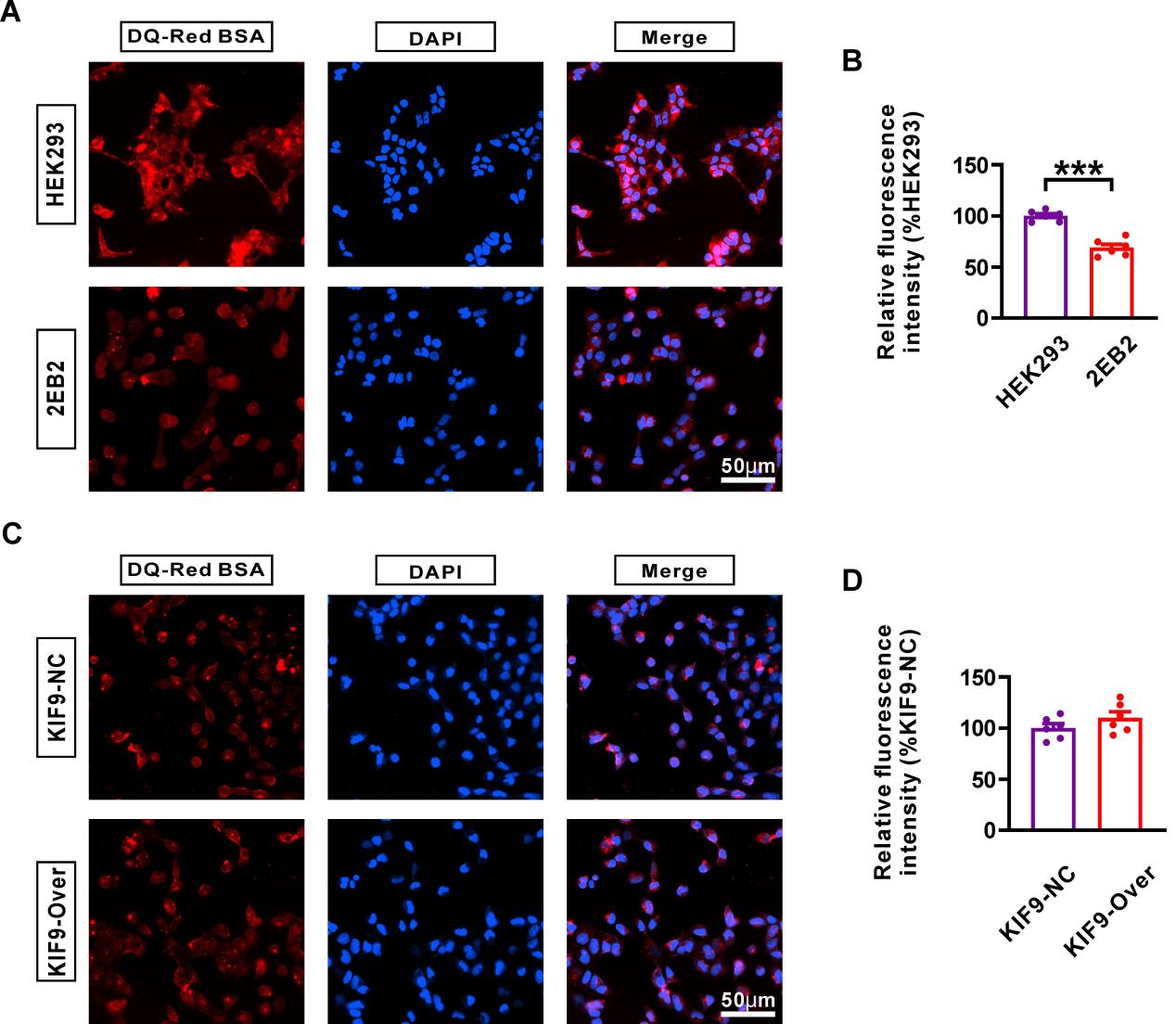


**Supplementary Fig. 4 Overexpression of KIF9 did not affect lysosomal degradation capability.** (A) DQ-Red BSA was used to detect the lysosomal degradation capability of HEK293 and 2EB2 cells. (B) The analysis was conducted in six randomly selected fields and the relative fluorescence intensity of DQ-Red BSA was quantified with ImageJ software. Scale bar: 50 μm. n=6 in each group. ***p<0.001 by unpaired Student’s t-test. (C) DQ-Red BSA was used to evaluate the lysosomal degradation capability of 2EB2 cells transfected with KIF9-NC or overexpressing KIF9 plasmid. (D) The analysis was conducted in six randomly selected fields and the relative fluorescence intensity of DQ-Red BSA was quantified with ImageJ software. Scale bar: 50 μm. n=6 in each group.

**
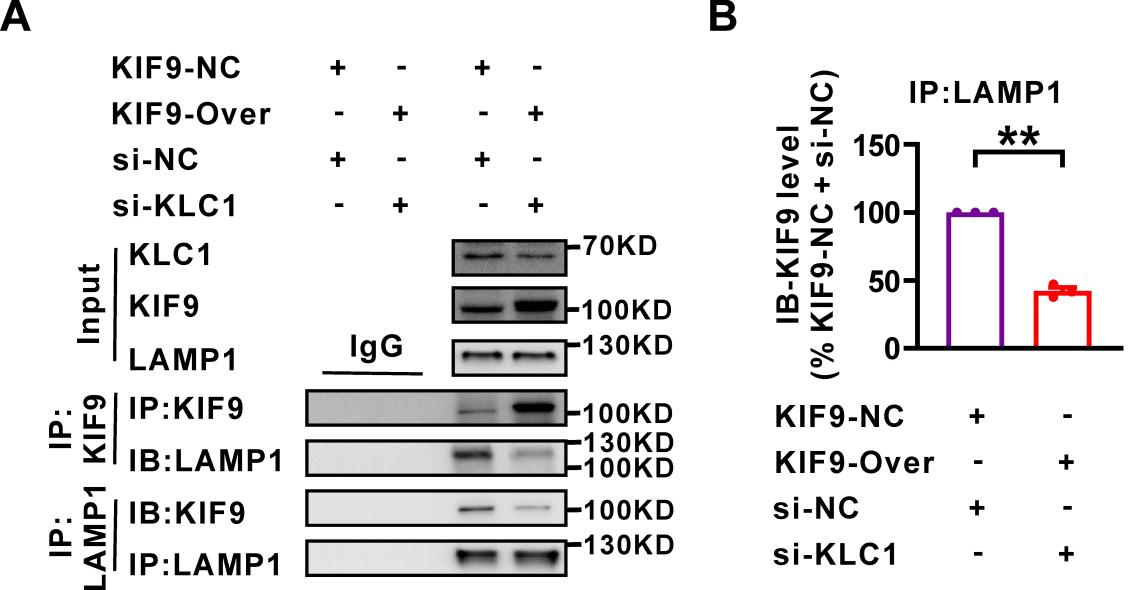
**

**Supplementary Fig. 5 Overexpression of KIF9 failed to restore the reduced interaction between KIF9 and LAMP1 caused by KLC1 knockdown.** (A-B) Interaction between KIF9 and LAMP1, and LAMP1 protein levels obtained through KIF9 interaction following the co-transfected KIF9-Over and si-KLC1 into the HEK293 cells, evaluated by Co-IP. n=3 in each group. **p<0.001 by unpaired Student’s t-test.
